# Nutritional status and extended metabolic screening in Egyptian children with uncomplicated type 1 diabetes

**DOI:** 10.1038/s41598-024-70660-8

**Published:** 2024-09-10

**Authors:** Hala M. Sakhr, Mohammed H. Hassan, Ahmed El-Abd Ahmed, Nagwan I. Rashwan, Rehab H. Abdel-Aziz, Amr S. Gouda, Rana Toghan

**Affiliations:** 1 Department of Pediatrics, Qena Faculty of Medicine, South Valley University, Qena, Egypt; 2 Department of Medical Biochemistry, Faculty of Medicine, South Valley University, Qena, Egypt; 3 Department of Medical Physiology, Faculty of Medicine, South Valley University, Qena, Egypt; 4 Department of Biochemical Genetics, Human Genetics and Genome Research Institute, National Research Centre, Giza, Egypt

**Keywords:** Metabolic screening, Nutritional status, T1DM, Glycemic control, Endocrinology, Health care

## Abstract

Nutritional status assessment, including amino acids, carnitine, and acylcarnitine profile, is an important component of diabetes care management, influencing growth and metabolic regulation. A designed case–control research included 100 Egyptian participants (50 T1DM and 50 healthy controls) aged 6 to 18 years old. The participants' nutritional status was assessed using the Body Mass Index (BMI) Z-score. Extended metabolic screening (EMS) was performed using a high-performance liquid chromatography-electrospray ionization-mass spectroscopy system to evaluate the levels of 14 amino acids, free carnitine, and 27 carnitine esters. T1DM children had considerably lower anthropometric Z-scores than the control group, with 16% undernutrition and 32% short stature. Total aromatic amino acids, phenylalanine, phenylalanine/tyrosine ratio, proline, arginine, leucine, isoleucine, free carnitine, and carnitine esters levels were considerably lower in the diabetic group, suggesting an altered amino acid and carnitine metabolism in type 1 diabetes. BMI Z-score showed a significant positive correlation with Leucine, Isoleucine, Phenylalanine, Citrulline, Tyrosine, Arginine, Proline, free carnitine, and some carnitine esters (Acetylcarnitine, Hydroxy-Isovalerylcarnitine, Hexanoylcarnitine, Methylglutarylcarnitine, Dodecanoylcarnitine, Tetradecanoylcarnitine, and Hexadecanoylcarnitine). HbA1c% had a significant negative correlation with Total aromatic amino acids, Branched-chain amino acid/Total aromatic amino acids ratio, Glutamic Acid, Citrulline, Tyrosine, Arginine, Proline, and certain carnitine esters (Propionylcarnitine, Methylglutarylcarnitine, Decanoylcarnitine, Octadecanoylcarnitine and Octadecenoylcarnitine), suggest that dysregulated amino acid and carnitine metabolism may be negatively affect the glycaemic control in children with TIDM. In conclusion, regular nutritional assessments including EMS of T1DM patients are critical in terms of diet quality and protein content for improved growth and glycemic management.

## Introduction

Diabetes mellitus (DM) is a complex metabolic condition defined by chronic hyperglycemia caused by abnormalities in insulin production, action, or both. Poor insulin secretion and/or diminished tissue insulin responses cause inefficient insulin action on target tissues, resulting in glucose, lipid, and protein metabolic problems^1^.

Pediatric undernutrition is the disparity between nutrient need and consumption, resulting in an accumulative deficiency in protein, micronutrients, and energy which can have a deleterious impact on normal growth and development^2^.

Nutritional disorders such as obesity and chronic undernutrition have been connected with diabetes mellitus^3,4^. Children with type 1 diabetes should have their nutritional status examined frequently because they are in a growing stage and liable to malnutrition owing to the chronic and burdensome nature of the condition or the concomitant occurrence of related autoimmune diseases such as celiac disease^5,6^.

The human body's protein metabolism is a continuous process that includes both protein breakdown and protein synthesis. Lean body mass can be increased or lost as a result of changes in either of these processes, or both. Braziuniene et al.,^7^ in their study concluded that insulin's primary anabolic effect on protein metabolism in adolescents with type 1 diabetes is to prevent whole-body proteolysis with no evidence of insulin's ability to increase protein synthesis in these adolescent participants, even when they were adequately fed.

Abnormal amino acid metabolism impairs the body's balance, preventing growth and development. Furthermore, amino acids have a role in gene expression, hormone synthesis and secretion, food metabolism, oxidative stress protection, immunity, reproduction, growth, and child development^8^.

Carnitine, a water-soluble tiny molecule similar to a vitamin, transports long-chain fatty acids from the cytosol to the mitochondria for β-oxidation and energy production. L-carnitine has been proven to have antioxidant and anti-inflammatory characteristics, as well as increasing membrane stability, insulin sensitivity, dyslipidemia, and protein nutrition^9^.

Is there a link between amino acid plasma levels, carnitine, and acylcarnitines, nutritional status, and glycemic control in Type 1 diabetes patients, and are there any differences when compared to healthy controls? So the rationale and the target of this study is to analyze these metabolomics and to assess their relationship with the nutritional status of type 1 diabetic patients as well as indicators of glycemic control to help us understand the current situation of our cases and better address, treat, and improve quality of life in those vulnerable patients.

## Patients and methods

### Study design and participants

Based on the standards established in the Declaration of Helsinki, case–control research was conducted with 100 Egyptian children and adolescents aged 6–18 years, with 50 diagnosed with type 1 diabetes^10^ and 50 healthy sex and age-matching control groups during the study period from January 2023 to December 2023. They were chosen from the outpatient pediatric endocrinology clinics of Qena University Hospitals, South Valley University, Upper Egypt, following permission from Qena University's ethics committee with an ethical approval code (SVUMEDPED02542212532). A formal agreement was obtained from participants above the age of 16 and all children's caregivers under the age of 16.

Cases with concomitant chronic illness, chronic diabetes complications, cases associated with autoimmune disease, or vegetarian patients were all eliminated from the analysis.

The sample size was set using the proportion difference approach level to ensure 80% power and 95% confidence level in the significance (type 1 error), control-to-case ratio of 1:1, and supposed odds ratio of ≥ 2^11^. So at least 48 participants should be presented in each group and we include 50 patients and 50 controls.

### Clinical evaluation of the included children

Precise medical data were obtained from all participants including age, gender, residence, nutritional history, family history of type 1 diabetes, age of diabetic onset, history of recurrent diabetic ketoacidosis^12^, history of recurrent unexplained hypoglycemia, and an inquiry about type and doses of injected insulin was obtained. Anthropometric evaluations were completed and plotted correctly for patients wearing light clothing and without shoes. Using a conventional stadiometer, height was measured to the closest 0.1 cm, and an electronic scale was used to determine weight to the closest 0.1 kg. Body mass index (BMI) was computed as kg/m^2^. The participants' nutritional status was evaluated using World Health Organisation (WHO) standard techniques for children aged 5 to 19 years^13^. The BMI was transformed to Z-scores using WHO AnthroPlus 1.0 and classed as obese (> + 2SD), overweight (≥ 1 to + 2SD), normal (≥ − 2 to ≤  + 1SD), and underweight (< − 2 SD). The weight-for-age Z-score (WAZ) and height-for-age Z-score (HAZ) are derived by subtracting the observed value from the reference population's median value and dividing it by its standard deviation^14^. Short stature was considered with a height that was ≥ 2 standard deviations lower than the mean for children of the same gender and chronological age^15^.

Complete systematic respiratory, cardiac, abdominal, and neurological evaluation was performed for the studied groups.

### Laboratory workup

A 4 mL of venous blood was obtained from each participant into an EDTA tube for the laboratory evaluation after a 12-h fast. HbA1c was measured using a Cobas c311 (provided by Hitachi, Roche Diagnostics, Germany). A normal HbA1c (*glycated hemoglobin*) result should be 4.5–5.6%, and children with T1DM should have a level less than 7.5% to be considered well-controlled^16^. Plasma-free amino acids, carnitines (CN), and acylcarnitines (AcylCNs) were determined using a high-performance liquid chromatography-electrospray ionization-mass spectroscopy (HPLC–ESI–MS) system (supplied by Waters Company, England) as described in a previously published work^17^. Briefly, The amino acid assays were performed using stable isotope internal standards (ISTDs) from Cambridge Isotope Laboratories. A total of 14 amino acids were measured (Leucine, Isoleucine, Methionine, Phenylalanine, Valine, Alanine, Aspartic acid, Glutamic acid, Citrulline, Ornithine, Tyrosine, Arginine, Glycine, and Proline) and classified as essential, non-essential, and conditionally essential amino acids^18^. Phenylalanine/tyrosine ratio, Total aromatic amino acids "AAA" (Tyrosine + Phenylalanine), Total branched-chain amino acids "BCAA"(Valine + Leucine + Isoleucine), and branched-chain amino acids (BCAA)/aromatic amino acids (AAA) ratios were calculated. Also, a total of 28 carnitine levels were assessed. Free L-carnitine is referred to as C0 or FC, while acylcarnitines were divided into three classes^19^, based on the number of carbon atoms in the acyl group chain: short-chain acylcarnitines, medium-chain acylcarnitines, and long-chain acylcarnitines.

### Statistical analysis

Data input and analysis were conducted with Statistical Package for Social Science version 22. Data were tested for normality using Kolmogorov–Smirnov and Shapiro– Wilk tests and were found to be normally distributed. Numbers, percentages, means, and standard deviations were employed to present the information. Chi-squared and student t-tests were utilized. Pearson's correlation coefficient was employed. statistical significance was defined as a p-value < 0.05.

### Ethics approval and consent to participate

Cases were collected correspondingly with the guidelines established in the Declaration of Helsinki. This study was approved by the ethics committee of South Valley University (Ethical approval code: SVUMEDPED02542212532). Informed written consent was taken from participants ≥ 16 years old and from the parents of the younger participants for participation in the study and publication.

## Results

### Clinical data of the participants

This study included 50 patients with T1DM with a mean age of 12.58 years ± 2.5; females represent the major percentage 54.0%. Cases compared to 50 age- and sex-matched healthy children as a control group, with a mean age of 12.54 years ± 2.0; 25 males (50.0%) and 25 females (50.0%). Comparing the two groups, there are no significant differences concerning age and sex, but significantly lower values were observed as regards anthropometric data Z-scores (weight, height, and MBI Z-scores) among the included TIDM children with 16 cases (32%) having short stature, 8 cases (16%) having undernutrition, and 2 cases (4%) had overweight **(**Table 1**)**, indicating a high prevalence of malnutrition among T1DM patients.

**Table 1:** Characteristics data of the participants.

Variables (mean ± SD)	T1DM (N = 50)	Controls (N = 50)	P-value
Age (years)	12.58 ± 2.5	12.54 ± 2.0	0.93
Sex: Males (N.%)	23 (46.0%)	25(50%)	0.688
Females (N.%)	27 (54.0%)	25 (50%)	
Age at diabetic onset (years)	5.10 ± 3.0	–	–
Diabetic duration (years)	6.83 ± 3.11	–	–
Insulin dose U/Kg	1.19 ± 0.24	–	–
Weight (Kg)	36.3 ± 11.5	43.8 ± 9.06	0.0005**
Weight-for-age Z-score	−1.29 ± 1.4	−0.02 ± 0.45	0.0001**
Height (cm)	140.98 ± 13.6	151.4 ± 9.8	0.0001**
Height-for-age Z-score	−1.65 ± 1.4	−0.24 ± 0.76	0.0001**
BMI (kg/m^2^)	17.8 ± 2.96	18.8 ± 2.18	0.057
BMI-for-age Z-score	−0.49 ± 1.3	0.146 ± 0.46	0.0015*
HbA1c (%)	9.87 ± 1.13	4.14 ± 0.63	0.0001**
Random blood glucose (mg/dl)	379.30 ± 87.13	95.66 ± 11.34	0.0001**

BMI: Body Mass Index. Hb A1c: *Hemoglobin A1C(*glycated hemoglobin)*.* * Statistically significant predictor (p < 0.05). ** Highly Statistically significant predictor (p < 0.001).

### Laboratory data of the included studied groups

Evaluation of the levels of Plasma amino acid profiles and some of their derivatives among the study groups showed that Children with T1DM had significantly lower levels of Leucine & Isoleucine (146.70 ± 29.48 μmol/l), Phenylalanine (57.90 ± 5.79 μmol/l), Phenylalanine/Tyrosine ratio (0.57 ± 0.03), Total aromatic amino acids "AAA" (158.62 ± 13.32 μmol/l), Arginine (21.68 ± 4.31 μmol/l), and Proline (161.0 ± 58.10 μmol/l) compared to the control group with p-Value < 0.05 as shown in Table 2, indicating deficiency of some amino acids (most of them are essential amino acids that can’t be supplied by the human body and should be supplied in their diet) among T1DM children.

**Table 2:** Plasma-free amino acid levels among the study groups.

PFAAs (μmol/l) (mean ± SD)	T1DM (N = 50)	Control (N = 50)	P- value
Leucine & Isoleucine	146.70 ± 29.48	166.44 ± 30.68	0.0014**
Methionine	28.07 ± 8.82	26 ± 5.074	0.153
Phenylalanine	57.90 ± 5.79	68.67 ± 12.27	0.0001**
Phenylalanine/tyrosine ratio	0.57 ± 0.03	0.67 ± 0.11	0.0001**
Valine	133.13 ± 36.56	119.72 ± 41.25	0.088
Total branched-chain amino acids	279.82 ± 48.37	286.167 ± 49.58	0.518
Total aromatic amino acids	158.62 ± 13.32	170.56 ± 16.19	0.0001**
BCAA/AAA ratio	1.76 ± 0.26	1.676 ± 0.26	0.109
Alanine	317.40 ± 100.04	336.68 ± 65.50	0.257
Aspartic acid	178.80 ± 60.01	165.44 ± 28.49	0.158
Glutamic acid	102.22 ± 17.64	103.89 ± 26.10	0.708
Citrulline	23.30 ± 4.83	25.34 ± 6.67	0.083
Ornithine	113.68 ± 26.16	108.57 ± 23.30	0.304
Tyrosine	100.72 ± 8.09	101.89 ± 6.79	0.435
Arginine	21.68 ± 4.31	24.33 ± 4.33	0.002*
Glycine	214.52 ± 76.45	235.44 ± 20.46	0.053
Proline	161.0 ± 58.10	198.42 ± 41.83	0.0004**

BCAA/AAA ratio: Branched-chain amino acids/aromatic amino acids. -PFAAs: plasma-free amino acids. * Statistically significant predictor (p < 0.05). ** Highly statistically significant predictor (p < 0.001).

The current study found that children with T1DM exhibited significantly lower levels of free carnitine (41.48 ± 11.30 μmol/l) compared to (49.44 ± 11.06) in the control group. Short-chain acylcarnitines levels revealed also that T1DM had lower values versus the control group for Propionylcarnitine, Malonylcarnitine, Butyrylcarnitine, Hydroxybutyrylcarnitine, Methylcrotonylcarnitine, Hydroxy-Isovalerylcarnitine, Glutarylcarnitine, and Hexanoylcarnitine with p-Value < 0.05. Medium-chain acylcarnitines Decenoylcarnitine, Dodecanoylcarnitine, Hydroxytetradecanoylcarnitine, and Long-chain acylcarnitines Hexadecanoylcarnitine, Hexadecenoylcarnitine, Hydroxyhexadecanoylcarnitine, Octadecenoylcarnitine, Hydroxyoctadecanoylcarnitine, and Hydroxyoctadecenoylcarnitine had also significantly lower levels than the control group (Table 3), indicating that dysregulated lipid metabolism in T1DM could be related to a lack of plasma carnitine and some of its esters among those patients.

**Table 3:** Levels of carnitine and acylcarnitines among the study groups.

Variables (mean ± SD) (μmol/l)	T1DM (N = 50)	Control (N = 50)	P- value
Free carnitine (C0)	41.48 ± 11.30	49.44 ± 11.06	0.0006**
*Short-chain acylcarnitines*			
Acetyl carnitine (C2)	47.34 ± 11.77	46.77 ± 11.15	0.804
Propionylcarnitine (C3)	3.54 ± 1.13	4.17 ± 1.29	0.01*
Malonylcarnitine(C3DC)	0.13 ± 0.05	0.18 ± 0.04	0.0001**
Butyrylcarnitine (C4)	0.74 ± 0.35	1.15 ± 0.35	0.0001**
Hydroxybutyrylcarnitine (C4OH)	0.38 ± 0.24	0.59 ± 0.097	0.0001**
Methylmalonylcarnitine (C4DC)	1.61 ± 0.12	1.69 ± 0.48	0.255
Methylcrotonylcarnitine (C5:1)	0.24 ± 0.10	0.34 ± 0.05	0.0001**
Isovalerylcarnitine (C5)	1.01 ± 0.14	1.09 ± 0.297	0.088
Hydroxy-Isovalerylcarnitine (C5OH)	0.36 ± 0.11	0.53 ± 0.13	0.0001**
Glutarylcarnitine (C5DC)	0.11 ± 0.05	0.16 ± 0.04	0.0001**
Hexanoylcarnitine (C6)	0.26 ± 0.07	0.32 ± 0.08	0.0001**
Methylglutarylcarnitine (C6DC)	0.14 ± 0.04	0.15 ± 0.04	0.214
*Medium-chain acylcarnitines*			
Octanoylcarnitine (C8)	0.20 ± 0.03	0.21 ± 0.05	0.228
Decanoylcarnitine (C10)	0.24 ± 0.05	0.23 ± 0.05	0.315
Decenoylcarnitine (C10:1)	0.15 ± 0.04	0.21 ± 0.06	0.0001**
Dodecanoylcarnitine (C12)	0.40 ± 0.11	0.49 ± 0.03	0.0001**
Dodecenoylcarnitine (C12:1)	0.07 ± 0.01	0.07 ± 0.03	1.000
Tetradecanoylcarnitine (C14)	0.72 ± 0.15	0.67 ± 0.23	0.209
Tetradecenoylcarnitine (C14:1)	0.46 ± 0.08	0.47 ± 0.14	0.662
Hydroxytetradecanoylcarnitine (C14OH)	0.15 ± 0.06	0.23 ± 0.04	0.0001**
*Long-chain acylcarnitines*			
Hexadecanoylcarnitine (C16)	4.63 ± 2.76	5.95 ± 1.97	0.007*
Hexadecenoylcarnitine (C16:1)	0.31 ± 0.04	0.38 ± 0.13	0.0004**
Hydroxyhexadecanoylcarnitine (C16OH)	0.15 ± 0.03	0.17 ± 0.05	0.017*
Octadecanoylcarnitine (C18)	2.05 ± 0.43	2.03 ± 0.87	0.884
Octadecenoylcarnitine (C18:1)	1.79 ± 0.38	2.02 ± 0.47	0.008*
Hydroxyoctadecanoylcarnitine (C18OH)	0.21 ± 0.06	0.27 ± 0.09	0.0002**
Hydroxyoctadecenoylcarnitine (C18:1OH)	0.19 ± 0.04	0.24 ± 0.05	0.0001**

* Statistically significant predictor (p < 0.05). ** highly Statistically significant predictor (p < 0.001).

### Correlation between plasma free amino acids (PFAAs) with HbA1c% and BMI-Z score in diabetic patients

A significant negative correlation was figured out between HBA1c% and the following amino acids; Total aromatic amino acids "AAA", BCAA/AAA ratio, Glutamic Acid, Citrulline, Tyrosine, Arginine, and Proline. BMI Z-score positively correlated with Leucine, Isoleucine, Phenylalanine, Citrulline, Tyrosine, Arginine, and Proline Table 4**,** indicating the possible contributory role of some amino acids availability with the nutritional status and the degree of glycemic control among children with T1DM.

**Table 4:** Plasma free-amino acids correlation with HbA1c% and BMI-Z-score.

PFAAs	HbA1c	
Total aromatic amino acids "AAA"	R-value	−0.359*
	P-value	0.011
BCAA/AAA ratio	R-value	−0.279*
	P-value	0.05
Glutamic Acid	R-value	−0.332*
	P-value	0.019
Citrulline	R-value	−0.604**
	P-value	0.000
Tyrosine	R-value	−0.477**
	P-value	0.000
Arginine	R-value	−0.660**
	P-value	0.000
Proline	R-value	−0.325*
	P-value	0.021
PFAAs	**BMI Z-score**	
Leucine and Isoleucine	R-value	0.961**
	P-value	.001
Phenylalanine	R-value	0.735**
	P-value	.000
Citrulline	R-value	0.595**
	P-value	.006
Tyrosine	R-value	0.744**
	P-value	0.000
Arginine	R-value	0.548*
	P-value	0.012
Proline	R-value	0.666**
	P-value	0.001

*Correlation is significant at the 0.05 level (2-tailed). **Correlation is significant at the 0.01 level (2-tailed). – BMI Z-score: Body Mass Index Z-score.—Hb A1c: *Hemoglobin A1C* glycated hemoglobin*.—*BCAA/AAA ratio: Branched-chain amino acids/aromatic amino acids.- – PFAAs: plasma-free amino acids.

### Correlation between carnitine and acylcarnitines derivatives with HbA1c% and BMI Z-score in T1DM

A significant negative correlation was detected between HBA1c % and the following derivatives; Propionylcarnitine, Methylglutarylcarnitine, Decanoylcarnitine, Octadecanoylcarntine, and Octadecenoylcarnitine. BMI Z-score had a significant positive correlation with Free carnitine, short-chain acylcarnitines (Acatylcarnitine, Hydroxy-Isovalerylcarnitine, Hexanoylcarnitine, and Methylglutarylcarnitine), Medium-chain acylcarnitines (Dodecanoylcarnitine, and Tetradecenoylcarnitine), and long-chain acylcarnitines Hexadecenoylcarnitine (C16) as shown in Table. 5**.** Thus carnitine and some of its esters are significantly linked to the nutritional status and glycemic control among diabetic children.

**Table 5:** Carnitine and acylcarnitines derivatives correlation with HbA1c% and MBI Z-score in T1DM.

Short-chain acylcarnitines	HbA1c%	
Propionylcarnitine(C3)	R-value	−0.413**
	P-value	0.000
Methylglutarylcarnitine (C6DC)	R-value	−0.474**
	P-value	0.001
**Medium-chain acylcarnitines**		
Decanoylcarnitine (C10)	R-value	−0.442**
	P-value	0.001
***Long-chain acylcarnitines***		
Octadecanoylcarntine (C18)	R-value	−0.229*
	P-value	0.035
Octadecenoylcarnitine (C18:1)	R-value	−0.497**
	P-value	0.000
	**BMI Z-score**	
**Free Carnitine (C0)**	R-value	0.659**
	P-value	0.002
**Short-chain acylcarnitines**		
Acatylcarnitine (C2)	R-value	0.842**
	P-value	0.000
Hydroxy-Isovalerylcarnitine (C5-OH)	R-value	0.811**
	P-value	0.000
Hexanoylcarnitine (C6)	R-value	0.693**
	P-value	0.001
Methylglutarylcarnitine (C6DC)	R-value	0.664**
	P-value	0.001
**Medium-chain acylcarnitines**		
Dodecanoylcarnitine (C12)	R-value	0.890**
	P-value	0.000
Tetradecenoylcarnitine (C14)	R-value	0.741**
	P-value	0.000
**long-chain acylcarnitines**		
Hexadecenoylcarnitine (C16)	R-value	0.744**
	P-value	0.000

*Correlation is significant at the 0.05 level (2-tailed).- **Correlation is significant at the 0.01 level (2-tailed). – BMI Z-score: Body Mass Index Z-score.—Hb A1c: *Hemoglobin A1C (*glycated hemoglobin)*.*

## Discussion

Nutritional management is a key component of diabetes care and education. Different countries have vastly different cultures and socioeconomic positions, which impact and dominate eating patterns. Although the strong evidence for nutritional needs in diabetic children, the scientific evidence basis for many elements of diabetic nutritional management is still developing^20^.

Despite improvements in medical care and therapy for T1DM children, their growth is still suboptimal, which is most likely due to persistent metabolic dysregulation^21^. The current study demonstrated that children and adolescents with T1DM exhibited impaired nutritional status with weight, height, and MBI-Z scores significantly lower in comparison to the healthy group; 8 cases (16%) had undernutrition, 2 cases (4%) had overweight, and 16 cases (32%) having short stature. BMI Z-score showed a significant positive correlation with Tyrosine, Phenylalanine, Citrulline, Proline, Arginine, free carnitine, short-chain acylcarnitines (C2, C5-OH, C6, and C6DC), Medium-chain acylcarnitines (C12, and C14), and long-chain acylcarnitines C16.

Several investigations found that T1DM patients had considerably lower anthropometric measurements than the general population^22,23^. Hussein et al.^23^ found that 21.42% of children and adolescents with T1DM have wasting and 38.09% have short stature. Bizzarri et al.^24^ observed that reduced growth velocity, together with a decline in height standard deviation from the onset of diabetes to reach final adult height, has been a constant finding in T1DM, as both pre-pubertal growth velocity and the pubertal growth surge have been reported to be lower. Furthermore, Khadilkar et al.^25^ found 10.9% and 27.1% rates of wasting and short stature, respectively. Aljuhani et al.^26^ found 6.9% wasting and 11.9% short stature and Kayirangwa et al.^16^ found that stunting was prevalent in 30.9% of type 1 diabetic subjects they investigated. These variances may be attributed to changes in sample size and population variables between studies.

Plasma amino acid concentrations are tightly regulated by homeostatic mechanisms and their concentration changes have been reported in a variety of disorders, including metabolic diseases^27^ and malnutrition^28^.

The essential amino acids are derived from proteins involved in the growth process. Furthermore, the body cannot entirely or effectively synthesize its carbon skeleton de novo, thus it must be acquired by dietary intake to attain adequate sufficiency. Adequate energy intake and high-quality protein are two elements that promote linear growth in children^29^. Semba et al.^30^ discovered that stunted children had reduced serum concentrations of all nine essential amino acids, as well as non-essential amino acids (asparagine, glutamate, serine) and conditionally essential amino acids (arginine, glycine, glutamine).

In the current study, cases had an elevated HBA1c% (9.87 ± 1.13). Plasma amino acid profiles and some of their derivatives among the study groups revealed that children with T1DM had significantly lower levels of Phenylalanine, Phenylalanine/Tyrosine ratio, Total aromatic amino acids "AAA", Leucine & Isoleucine, Proline, and Arginine than the control group; suggesting an altered amino acid metabolism in type 1 diabetes. HBA1c levels were also found to be adversely connected with Tyrosine, Total aromatic amino acids, Branched-chain amino acid/Total aromatic amino acid ratio, Proline, Glutamic Acid, Arginine, and Citrulline.

Insulin deficit inhibits protein synthesis and promotes protein breakdown, hence amino acid metabolism may be changed in diabetes mellitus^31^. This may explain our findings about the high HBA1c% and its relationship to amino acid levels.

We found lower significant levels of free carnitine and carnitine esters levels in the diabetic group with HBA1c% negatively correlated with C3, C6DC, C10, C18, and C18:1. BMI Z-score positively correlated with free carnitine, C2, C5-OH, C6, C6DC, C12, C14, and C16.

Multiple authors have documented reduced plasma levels of L-carnitine in Type 1 diabetes patients^32,33^ and type 2 diabetes^34^. Morgane et al.^35^ discovered that free L-carnitine levels in individuals with newly diagnosed or long-term T1DM were considerably lower than in the healthy control group. La Marca^36^ and his colleagues found that levels of acyl-carnitines C2, C3, C5OH, C4, C16, and C18 were considerably lower in the cases compared to controls. Furthermore, the sum of the total measured amino acid concentrations tended to be lower in cases compared to controls.

L- carnitine is an important nutritional element supplemented in animal food as its endogenous synthesis is insufficient to satisfy metabolic needs, and a High-Fat Diet (HFD) can increase carnitine and its metabolite production^37^. Its supplementation enhances nitrogen balance by increasing protein formation or decreasing protein breakdown, preventing apoptosis, inhibiting inflammatory responses under pathological conditions, improves a variety of key pathways involved in pathological skeletal muscle loss^38,39^.

Diabetes disrupts glucose metabolism, resulting in a negative energy balance, especially during insulin shortage. A brief period of insulin deprivation affects mitochondrial function, protein synthesis, and enzyme activity^40^.

Multiple mechanisms have been proposed in support of the favorable effect of carnitine on glucose metabolism: it intensifies the mitochondrial oxidation of long-chain acyl-CoA, the aggregation of which would otherwise lead to insulin resistance in the muscle and heart; modifying the expression of glycolytic and gluconeogenic enzymes; adjusting the activity of the pyruvate dehydrogenase complex; provoking of the insulin-like growth factor-1 (IGF-1) axis; and IGF-1 signaling^41^.

Future studies are required to evaluate the effect of adequate nutrition treatment with particular essential amino acids & carnitine supplementation, when combined with other diabetes management strategies, can improve anthropometric parameters and glycemic control or not.

This study has some limitations as it was a single-center study with a small sample size and a lack of follow-up data on the included cases. Additional suggestions for performing a big multicenter study with a greater sample size to reinforce the study results. Despite this restriction, the fact that this study was conducted prospectively utilizing a standardized approach rather than a review of patients' files gives it a significant advantage.

## Conclusion

Determining dietary requirements and assessing the sufficiency of normal growth in minors with T1DM is a critical issue. T1DM patients had considerably lower anthropometric Z-scores than the control group, with 16% undernutrition and 32% short stature. The findings of the current study indicated lower levels of plasma amino acid levels (especially essential amino acids that can’t be synthesized in the human body and must be obtained through diet) and L-carnitine levels with significant association with lower anthropometric parameters and poor glycemic controls among the included participants with type 1 diabetes.

## Data Availability

The datasets used and/or analyzed during the current study are available from the corresponding author upon reasonable request, after obtaining the permission of our institute.
